# Use of alcohol vinegar in the inhibition of *Candida* spp. and its effect on the physical properties of acrylic resins

**DOI:** 10.1186/s12903-015-0035-5

**Published:** 2015-04-28

**Authors:** Ricardo Dias de Castro, Ana Carolina Loureiro Gama Mota, Edeltrudes de Oliveira Lima, André Ulisses Dantas Batista, Julyana de Araújo Oliveira, Alessandro Leite Cavalcanti

**Affiliations:** Graduate Program in Dentistry, Universidade Federal da Paraíba, Campus I, João Pessoa, Paraíba 58.051-900 Brazil; João Pessoa University Center, João Pessoa, Paraíba Brazil; Graduate Program in Dentistry, State University of Paraiba, Campina Grande, Paraíba Brazil

**Keywords:** Candidiasis, Oral, *Candida albicans*, Acetic acid, Dental prosthesis

## Abstract

**Background:**

Given the high prevalence of oral candidiasis and the restricted number of antifungal agents available to control infection, this study investigated the *in vitro* antifungal activity of alcohol vinegar on *Candida* spp. and its effect on the physical properties of acrylic resins.

**Methods:**

Tests to determine the Minimum Inhibitory Concentration (MIC) and Minimum Fungicidal Concentration (MFC) of vinegar alcohol (0.04 g/ml of acetic acid) and nystatin (control) were performed. The antifungal activity of alcohol vinegar was assessed through microbial growth kinetic assays and inhibition of *Candida albicans* adhesion to acrylic resin at different intervals of time. Surface roughness and color of the acrylic resin were analyzed using a roughness meter and color analyzer device.

**Results:**

Alcohol vinegar showed MIC_75%_ and MFC_62.5%_ of 2.5 mg/ml, with fungicidal effect from 120 min, differing from nystatin (p < 0.0001), which showed fungistatic effect. Alcohol vinegar caused greater inhibition of *C. albicans* adhesion to the acrylic resin (p ≤ 0.001) compared to nystatin and did not change the roughness and color parameters of the material.

**Conclusion:**

Alcohol vinegar showed antifungal properties against *Candida* strains and caused no physical changes to the acrylic resin.

## Background

Given the increased life expectancy and greater access of the population to dental care services, there is a higher prevalence of users wearing dentures due to significant tooth loss among the elderly, either in developed or developing countries [1,2].

Associated with the use of dental prostheses, the occurrence of infections caused by fungal species, particularly *Candida*, is common and mostly reported in individuals with poor general health and immunosuppression [3]. These infections are called denture stomatitis and can be clinically identified based on the different degrees of erythema present on the mucosa underlying the basis of partial or total prosthetic devices. Among the most common etiologic factors, are: poor oral hygiene; poorly fitted dentures; weakened immune system; indiscriminate use of antibiotics; and proliferation of *Candida* spp. [4].

One of the main factors contributing to the colonization of *Candida* spp. lies in their adaptability to a variety of growth “habitats” through formation of microbial communities attached to an extracellular polysaccharides matrix, including salivary proteins. *Candida* can quickly colonize the basis of the acrylic resin, providing greater stability for the fungal infection in the host [5].

As fungal infections of the oral cavity caused by *Candida* spp. are superficial, topical use of nystatin and miconazole has been recommended. In cases of no positive response to these therapeutic agents, other substances such as fluconazole and ketoconazole may be prescribed for systemic use [6,7]. However, the indiscriminate use of conventional antifungal agents results in the selection of resistant strains, especially in immunosuppressed patients and in those with severe systemic diseases [8]. This fact justifies the development of new therapies for use in daily clinical practice [9]. It should also be mentioned that many *Candida* spp. are able to penetrate the acrylic resin used in the manufacture of prosthetic devices at depths ranging from 1 to 2 μm [10], thus highlighting the need for a product that allows removing the biofilm without harming the mechanical properties of the resin. It is worth noting that among the required properties of materials used in the manufacture of prostheses, those related to roughness, surface tension, electrostatic interactions and hardness are of clinical importance, since they may influence biofilm accumulation and color change. Surface roughness causes adhesion and retention of *C. albicans*, which is of particular importance for the onset of stomatitis [11].

In this background, it is hypothesized that alcohol vinegar can control and prevent denture stomatitis due to its disinfectant action on the acrylic resin used in the manufacture of dentures. A low hydrogen potential leading to diffusion of acetic acid and its possible interaction with enzymes involved in the formation of ergosterol, a major component of fungal plasma membranes, may explain the known antimicrobial activity of alcohol vinegar, especially anti-*Candida* properties. Furthermore, it has low cost and easy access [12,13]. This study aimed to evaluate the *in vitro* antifungal effects on alcohol vinegar on *Candida* spp., and to verify its effect on the physical properties of acrylic resin as to surface roughness and color change.

## Methods

Alcohol vinegar brand Minhoto® batch L281D (Ind. Reunidas Raymundo da Fonte S.A, Paulista, PE, Brazil) was used as the test product, containing 4% acetic acid (0.04 g/ml) in its composition. The product was tested for the Minimum Inhibitory Concentration (MIC), Minimum Fungicidal Concentration (MFC), microbial growth kinetics, inhibition of *Candida* spp. adhesion to the surface of acrylic resin, and effects on surface roughness and color parameters. Nystatin (Sigma – Aldrich Brasil, São Paulo, SP, Brazil) was used as a positive control. During the tests, controls for yeast viability were also performed.

The following strains were used: *Candida albicans* ATCC 76485, *Candida albicans* LM 21, *Candida albicans* MI03, *Candida albicans* LM 615, *Candida albicans* LM 13, *Candida tropicalis*, ATCC 13803, *Candida tropicalis* LM 33 and *Candida tropicalis* LM 70. Colonies were suspended in 5 ml of sterile saline, 0.145 mol/L (0.85% NaCl). The resulting suspension was placed in a vortex mixer (Phoenix ®) for 15 seconds, and cell density was adjusted using a spectrophotometer to 0.5 McFarland scale at a wavelength of 530 nm.

### Antimicrobial activity (MIC and MFC)

MIC was determined by the microdilution technique using 96-well microplates [14]. A total of 100 μl of 2-fold concentrated Sabourand Dextrose Broth (SDB) (Difco Laboratories, Detroit, Mich., USA) was distributed in each well, followed by 100 μl of test substance (alcohol vinegar or nystatin at initial concentrations of 40,000 μg/ml and 100 μg/ml, respectively). An aliquot of 100 μl was collected from the first well and then dispensed into the following one, in order to proceed with a 2-fold serial dilution. Approximately 10 μl of inoculum were dispensed into each well. Tests were performed in triplicate and plates were incubated at 35°C for 48 hours. MIC was considered as the lowest concentration able to inhibit visible growth of the strains. In order to confirm the presence of viable or non-viable microorganisms, 10 μl of TTC dye ( 2,3,5 triphenyl tetrazolium chloride) was used, which reflects the activity of dehydrogenase enzymes involved in cellular respiration, staining live samples in red [15]. After 24 hours of incubation, visual reading was performed.

The MFC was determined after reading of MIC by collecting aliquots of 10 μl from the subcultures corresponding to MIC, MICx2 and MICx4 and mixing them to 100 μl of SDB in 96-well microplates. After incubation for 24 h at 35°C, visual reading was performed, considering the formation of cell clusters at the bottom of the wells [14]. For better clarity of results, 10 μl of TTC were added, and MFC was characterized as the lowest concentration of the test product able to inhibit growth of the strains [14].

#### Microbial growth kinetics

*C. albicans* LM 615 was selected for presenting the best microbial growth while obtaining MIC and MFC. For the assay, 0.5 ml of the yeast suspension was inoculated into 4.5 ml of SDB with the test substance (alcohol vinegar or nystatin) at concentrations adjusted to MIC, MICx2 and MICx4. At time intervals corresponding to 0 (t0), 30 (t1), 60 (t2), 120 (t3) and 180 minutes (t4), 10 μl aliquots were collected from the suspension and grown on Sabourand Dextrose Agar (SDA) (Difco Laboratories, Detroit, Mich., USA) plates. After incubation at 35°C for 24 h, the number of colony forming units (CFU) was counted. The results are presented as microbial death curves.

#### Preparation of acrylic resin specimens

In order to verify the inhibition of fungal adhesion to the acrylic resin, as well as changes in surface roughness and color, 78 circular specimens were made of auto-polymerized acrylic resin (Vip Flash ®, Vipi Dental Products, Pirassununga, São Paulo, Brazil), sizing 12 mm in diameter and 7 mm in thickness. The specimens were subjected to finishing with tungsten grinder (1508 Edenta AG, Haupistrasse, Switzerland), and polishing with carborundum sandpaper of different grit sizes (220, 330, 600 and 1200) and felt discs embedded in pumice stone paste/distilled water, followed by rinsing and sterilization.

#### Inhibition of fungal adhesion to the acrylic resin surface

Eighteen specimens were placed in test tubes containing 2.5 ml of SDB and 0.5 ml of yeast suspension (*C. albicans* LM 615) at 35°C for 48 h. The specimens were randomly divided into three groups: GI (n = 6) - Negative control (no antifungal substance, ensuring *C. albicans* adhesion to acrylic resin), GII (n = 6) - alcohol vinegar and GIII (n = 6) - nystatin.

Sufficient amounts of alcohol vinegar or nystatin solution were added in the tubes containing SDB/yeast suspension in GII and GIII groups, resulting in final concentrations corresponding to MIC, MICx2 and MICx4. After the incubation period, specimens were rinsed and placed in tubes containing 5 ml of saline (0.85% NaCl), and stirred for 60 s. Then, 10 μl of that solution were grown on SDA plates, which were incubated at 35°C for 48 h for further reading and bacterial counting, in triplicate.

#### Test of changes in surface roughness and color parameters of the acrylic resin

In the groups exposed to the effect of alcohol vinegar, concentrations corresponding to MIC (n = 6), MIC × 2 (n = 6) and MIC × 4 (n = 6) were used. Six specimens were exposed to nystatin and other six to no antifungal agent. Specimens were marked on the middle region, 1 mm to the right and 1 mm to the left, and submitted to a roughness meter device (SJ −201 Mitutayo – Japan, roughness parameter Ra: 0.8 *cut-off* mm) to perform the initial measurement (t = 0) at the three points. Surface roughness (Ra) was established as the mean f-values of the three points. Specimens were immersed in the solution for 30, 60, 120 and 180 minutes, washed with distilled water, dried on absorbent toweling and submitted to rugosimetric analysis.

With the purpose of measuring the color changes in the acrylic resin, 30 specimens were randomly divided into three groups, as previously mentioned. In the groups corresponding to alcohol vinegar and nystatin, the same concentrations as before were used. Specimens were exposed to the same time intervals (0, 30, 60, 120 and 180 minutes). The color analysis was determined by means of a color analyzer device (Model ACR-1023, Instrutherm Instrumentos de Medição Ltda, São Paulo, Brazil; Liquid crystal display of 59 mm × 34 mm; Measurement geometry: 45° / 0°; spectral range from 400 to 700 nm, color sensor of three photo transmitters of red, green and blue color), using the RGB system.

#### Statistical analysis

Data were recorded in a database on GraphPad Prism 2004. The antifungal activity was evaluated by two-way analysis of variance (ANOVA), followed by Tukey’s post-test, with significance level of 5%. To evaluate the changes caused by alcohol vinegar on the surface roughness and color of the acrylic resin, Kruskal-Wallis and Dunn’s post-test were used.

## Results

The MIC and MFC results of vinegar alcohol containing 0.04 g/ml of acetic acid, and nystatin (positive control) are shown in Table 1. All tested strains were found to be sensitive to the action of alcohol vinegar, with MIC_75%_ equal to 2500 μg/ml. *C. tropicalis* ATCC 13803 and *C. tropicalis* LM 33 were even more sensitive to alcohol vinegar, showing MIC of 1250 μg ml. However, these strains (25%) required higher concentrations of the test product to establish fungicidal activity, with MFC value of 10 mg/ml. The other strains (62.5%) showed MFC values equal to 2500 μg/ml, the same as the MIC. As to nystatin, all strains were inhibited at a concentration of 3.12 μg/ml, which was used as a control in the other tests.

**Table 1:** **MIC and MFC results of alcohol vinegar and nystatin on** ***Candida*** **species**

	**Alcohol vinegar**		**Nystatin**	
**Strains**	**MIC (**μ**g/mL)**	**MFC (**μ**g/mL)**	**MIC (**μ**g/mL)**	**MFC (**μ**g/mL)**
*C. albicans* LM 21	2500	2500	3.12	6.25
*C. albicans* MI03	2500	2500	3.12	12.5
*C. albicans* LM 615	2500	5000	3.12	12.5
*C. albicans* LM 13	2500	2500	3.12	3.12
*C. albicans* ATCC 76485	2500	2500	3.12	6.25
*C. tropicalis* ATCC 13803	1500	10000	3.12	12.5
*C. tropicalis* LM 33	1500	10000	3.12	3.12
*C. tropicalis* LM 708	2500	2500	3.12	3.12

**Figure 1:**
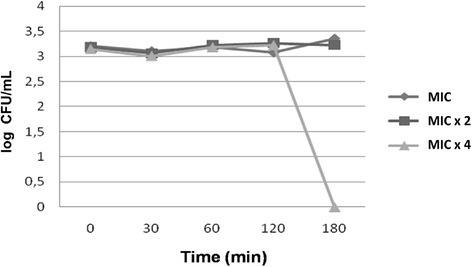
Microbial death curve versus time for alcohol vinegar at MIC, MIC × 2 and MIC × 4 concentrations.

Figure 1 shows the microbial death curves in the presence of alcohol vinegar at concentrations corresponding to MIC, MIC × 2 and MIC × 4 as a function of time. It was observed that for the first mentioned concentrations, there was fungistatic activity from 0 to 180 minutes. However, for MIC × 4, fungistatic activity of the test product was detected from 0 to 120 minutes, followed by fungicidal effects. The positive control (nystatin) at MIC, MIC × 2 and MIC × 4 showed fungistatic effects at all time intervals.

**Figure 2:**
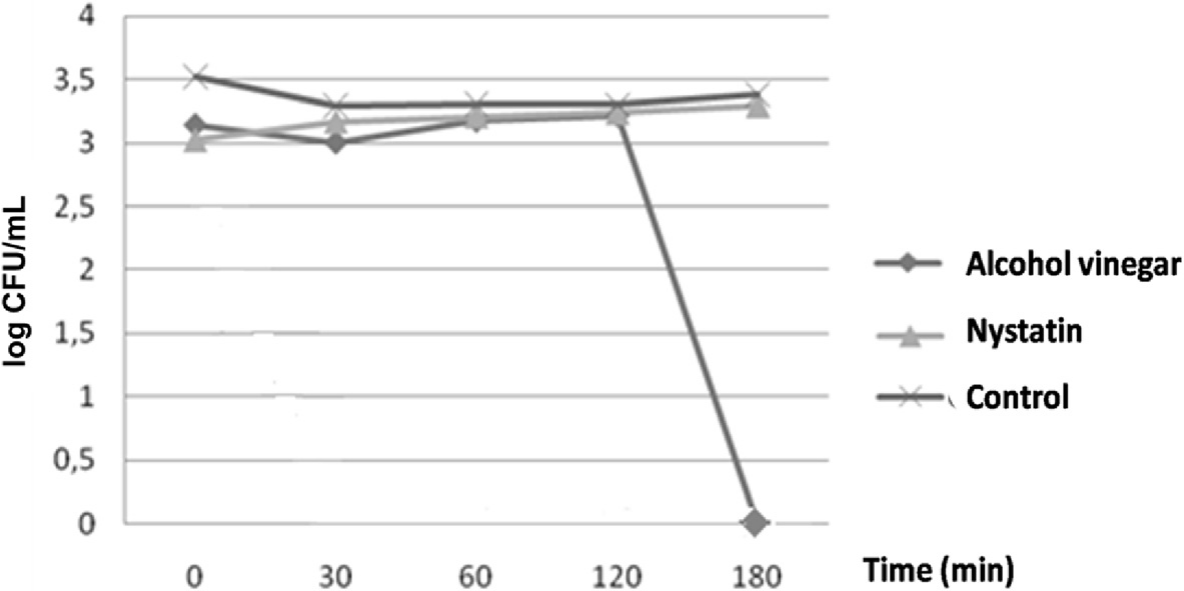
Microbial death *versus* time curve, showing the relationship between alcohol vinegar (acetic acid) and nystatin, used at MIC × 4, and control (absence of antifungal substance) (2 - Way ANOVA, p < 0.0001, Turkey , p <0.05).

Statistically significant difference was observed between the effect of alcohol vinegar and nystatin (p < 0.05), and also in relation to the negative control, represented by the absence of antifungal agent. This inference can be verified in Figure 2, particularly in the times 120 to 180 minutes, markedly the beginning of the fungicidal activity of alcohol vinegar.

**Figure 3:**
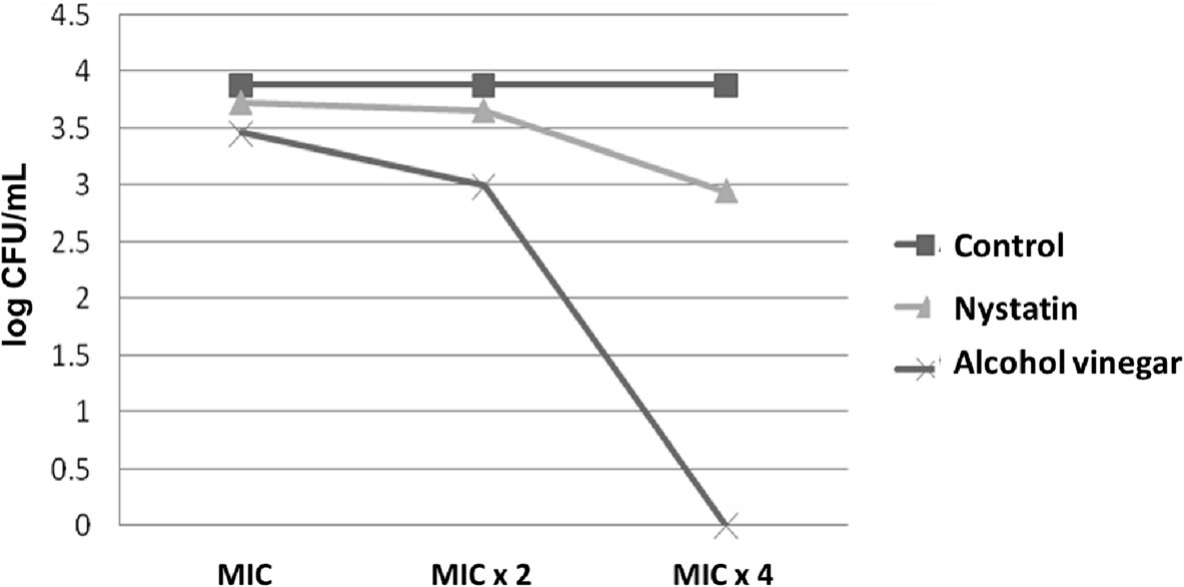
Adhesion test and CFU counting after the action of alcohol vinegar, nystatin and control (absence of antifungal substance).

Figure 3 shows data about the effectiveness of alcohol vinegar in the inhibition of the adhesion of *C. albicans* LM 615 to acrylic resin specimens, evidencing the action of acetic acid and nystatin (p < 0.05). The former was able to reduce microbial adhesion at MIC and MIC × 2, and completely prevent it at MIC × 4.

The surface roughness (Ra) analysis showed no significant change (greater than 0.2 μm) for specimens exposed to alcohol vinegar (Table 2).

**Table 2:** **Surface roughness (Ra) in** μ**m of specimens submitted to the action of alcohol vinegar over time**

**Time (min)**	**Alcohol vinegar**			**Nystatin**	**Control**
	**MIC**	**MIC ×2**	**MIC ×4**		
0	0.16	0.14	0.11	0.11	0.09
30	0.17	0.13	0.12	0.77	0.11
60	0.16	0.15	0.12	1.1	0.11
120	0.15	0.14	0.16	1.2	0.09
180	0.16	0.13	0.16	1.2	0.1

No significant difference between groups was observed (p > 0.05 - Kruskal Wallis Test).

With regard to possible changes in color, the results indicated that alcohol vinegar did not affect the color of exposed specimens at time intervals from 0 to 180 minutes at different concentrations, as seen in Table 3.

**Table 3:** **Color (values expressed in the RGB scale) of specimens after exposure to the action of alcohol vinegar**

	**Exposure time (min)**			**Nystatin**
	**0**	**60**	**120**	**180**
Alcohol vinegar MIC	2 2 4 1 6 9 (a) 1 4 4	(a)	(a)	(a)
Alcohol vinegar MIC *x*2	(a)	(a)	(a)	(a)
Alcohol vinegar MIC x4	(a)	(a)	(a)	(a)
Nystatin	(a)	(a)	(a)	(a)
Control	(a)	(a)	(a)	(a)

(a)Represents identical values.

## Discussion

The findings of our study indicate that alcohol vinegar has fungistatic and fungicidal effects on the tested strains of *Candida*. Previous studies have already verified the antifungal activity of vinegars [16], although MIC and MFC values are not expressed according to the microdilution technique, which is a low-cost and rapid method that provides reproducible results and requires small amounts of microbial suspension and culture media [14,17,18].

The mechanism of action of acetic acid, the main component of alcohol vinegar, is probably related to a reduced hydrogen potential, thus facilitating diffusion of the acid across the plasma membrane of fungal cells [18]. The literature has also reported inhibitory effect of acetic acid on 14α-lanosterol-demethylase, an important enzyme involved in the formation of ergosterol, which is essential for maintaining the integrity of the fungal plasma membrane [19,20].

Regarding nystatin, the MIC and MFC values obtained for all strains tested were in accordance with the literature [21]. Nystatin was chosen as control because it is a standard antifungal widely used for the topical treatment of fungal infections in the oral cavity [22]. Its mechanism of action is related to the inhibition of enzymes involved in the formation of ergosterol and, consequently, permeability of cell membrane [23].

Microbial growth kinetics is an important variable to evaluate the fungistatic or fungicidal activity of a particular substance, as well as to determine the influence of the exposure time on the cell death process [24]. The microbial kinetics of alcohol vinegar showed fungistatic activity at MIC and MICx2 for the time interval between 0 and 120 minutes, and from that time, there was fungicidal activity at MICx4 (10 mg ml). Fungicidal activity is considered as such when the product is able to reduce three logarithmic units to base 10, and when these changes are consistent with the fungistatic behavior.

Recognized as a dynamic process used to evaluate new antimicrobial agents, the microbial death *versus* time curve has not been mentioned in previous studies involving alcohol vinegar [25], hindering the standardization of results and the comparison between them. These studies have shown action times that appear to have been randomly adopted, ranging from 10 minutes to eight hours, with no methodological reference based on microbial kinetics [17,26].

Used as a standard antifungal, nystatin was also tested for the microbial death *versus* time curve. Previous studies have shown fungistatic activity of nystatin [23,27], corroborating the results of the present study, although fungicidal activity may be seen at higher concentrations.

During the selection of a disinfecting agent, one should evaluate its compatibility with oral tissues, as well as with materials that compose the basis of dental prostheses [28]. The adhesion of *Candida* spp. to the surface of acrylic resin is usually the first step in the colonization of tissues that come in contact with the removable denture, and tends to form a biofilm often resistant to conventional antifungal therapy [29]. *C. albicans* species are described as those with greater ability to adhere to oral mucosa cells and to the surface of the acrylic resin [30], which is why *C. albicans* was the selected species to determine MIC and MFC. Previous studies have shown that an undiluted alcohol vinegar solution was able to inhibit the adhesion of microorganisms, including *C. albicans,* to acrylic resin [31], which was also observed in this study. However, other investigations have shown that alcohol vinegar was not able to inhibit the adhesion of *C. albicans* cells to acrylic resin [32].

No significant changes were observed in the average surface roughness of the material, confirming previous findings [31,33]. Changes on surface roughness identified after exposure to different alcohol vinegar concentrations did not exceed the threshold value of 0.2 μm, above which influence of the surface roughness on the adhesion of microorganisms is expected, since surface irregularities serve as a microbiological niche, protecting infectious agents from the mechanical action of tooth brushing [34].

This study showed no color changes in the acrylic resin after a period of 180 minutes of exposure to alcohol vinegar and nystatin, confirming previous results [35]. The original color of a resin can be altered by the ingestion of large amounts of colorants, liquid absorption and immersion in disinfectant solutions that influence the surface roughness, damaging the aesthetics of the material [36]. A study using vinegars showed color change in acrylic resin after an interval from 12 to 30 days of immersion [31]. Even considering the importance of knowing the effect of prolonged exposure of acrylic resin to the action of disinfectants, shorter times such as those used in the present study must be used, since they simulate the time by which prosthetic devices are outside of the oral cavity. Previous studies have evaluated the effect of disinfectant solutions on the physical properties of acrylic resin. Ethanol, in varying concentrations, can produce effects on roughness and color [33]. The surface roughness of the acrylic resin was higher with the use of 3.8% sodium perborate and lower with 2% chlorhexidine gluconate [31].

The results of this test showed scientific evidence on the significant antifungal effect of alcohol vinegar against *Candida* species, as well as absence of negative influence on the acrylic resin. These findings support the possible use of the product, which should go through further laboratory studies investigating its effects on multi-species biofilm formation, microhardness of acrylic resin; clinical trials should also be considered to determine the safety, tolerability and clinical efficacy of this substance.

## Conclusion

Alcohol vinegar showed *in vitro* activity against *Candida* strains involved with denture stomatitis, with fungicide action after 120 minutes of exposure to a concentration of 10 mg/ml. It was also able to prevent the adhesion of *C. albicans* to acrylic resin and did not cause changes on the surface roughness and color of the acrylic resin after two hours of exposure.
